# Enhanced Nanoencapsulation of Sepiapterin within PEG-PCL Nanoparticles by Complexation with Triacetyl-Beta Cyclodextrin

**DOI:** 10.3390/molecules24152715

**Published:** 2019-07-26

**Authors:** Nataliya Kuplennik, Alejandro Sosnik

**Affiliations:** Laboratory of Pharmaceutical Nanomaterials Science, Department of Materials Science and Engineering, Technion-Israel Institute of Technology, 3200003 Haifa, Israel

**Keywords:** sepiapterin, triacetyl-β-cyclodextrin, hydrophilic drug/cyclodextrin complexes, spray-drying, methoxy-poly(ethylene-glycol)-poly(epsilon-caprolactone) nanoparticles

## Abstract

In this work, we aimed to improve the encapsulation efficiency of sepiapterin (SP), the natural precursor of the essential cofactor tetrahydrobiopterin (BH4) that displays mild water-solubility and a short biological half-life, within methoxy-poly(ethylene-glycol)-poly(epsilon-caprolactone)(mPEG-PCL) nanoparticles (NPs) by means of its complexation and hydrophobization with 2,3,6-triacetyl-β-cyclodextrin (TAβCD). For this, SP/TAβCD complexes were produced by spray-drying of SP/TAβCD binary solutions in ethanol using the Nano Spray Dryer B-90 HP. Dry powders were characterized by differential scanning calorimetry (DSC), Fourier-transform infrared spectroscopy (FTIR), and transmission and scanning electron microscopy (TEM and SEM, respectively) and compared to the pristine components and their physical mixtures (PMs). Next, SP was encapsulated within mPEG-PCL NPs by nano-precipitation of an SP/TAβCD complex/mPEG-PCL solution. In addition to the nano-encapsulation of a preformed complex within the polymeric NPs, we assessed an alternative encapsulation approach called drying with copolymer (DWC) in which pristine SP, TAβCD, and mPEG-PCL were co-dissolved in a mixture of acetone and methanol at the desired weight ratio, dried under vacuum, re-dissolved, and nano-precipitated in water. The dissolution-drying step was aimed to promote the formation of molecular hydrophobic interactions between SP, TAβCD, and the PCL blocks in the copolymer. SP-loaded mPEG-PCL NPs were characterized by dynamic light scattering (DLS) and SEM. NPs with a size of 74–75 nm and standard deviation (S.D., a measure of the peak width) of 21–22 nm were obtained when an SP:TAβCD (1:1 molar ratio) spray-dried complex was used for the nano-encapsulation and SEM analysis revealed the absence of free SP crystals. The encapsulation efficiency (*%EE*) and drug loading (*%DL*) were 85% and 2.6%, respectively, as opposed to the much lower values (14% and 0.6%, respectively) achieved with pristine SP. Moreover, the NPs sustained the SP release with relatively low burst effect of 20%. Overall, our results confirmed that spray-drying of SP/TAβCD solutions at the appropriate molar ratio leads to the hydrophobization of the relatively hydrophilic SP molecule, enabling its encapsulation within mPEG-PCL NPs and paves the way for the use of this strategy in the development of novel drug delivery systems of this vital biological precursor.

## 1. Introduction

**Figure 1:**
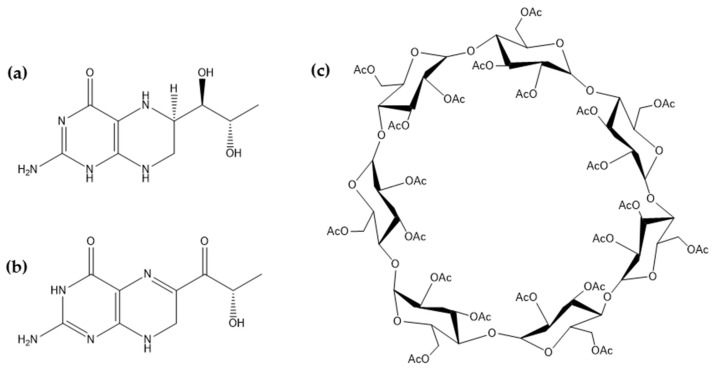
Chemical structure of (**a**) tetrahydrobiopterin (BH4), (**b**) sepiapterin (SP) and (**c**) 2,3,6-triacetyl-β-cyclodextrin (TAβCD).

Tetrahydrobiopterin (BH4, Figure 1a), a naturally occurring molecule, is present in most cells and tissues of higher organisms and it is a well-established as a cofactor in various essential enzymatic pathways that include the degradation of phenylalanine and the biosynthesis of neurotransmitters such as serotonin, melatonin, dopamine, noradrenaline, and adrenaline [1,2]. BH4 is also a key player in biological processes associated with cardiovascular homeostasis and the immune response [1,3]. Defects in BH4 metabolism caused by mutations in specific genes encoding for enzymes involved in its synthesis or regeneration (BH4 deficiency) lead to abnormally high phenylalanine levels in the blood and the deficiency of diverse neurotransmitters in the central nervous system (CNS) [4]. Decreased levels of BH4 have also been documented in neurological disorders such as Parkinson’s and Alzheimer’s disease, autism, and depression. In some of them, the administration of BH4 has been reported to improve the clinical symptoms [1,5]. However, BH4 undergoes fast aerobic degradation, which results in a decrease in treatment efficacy [6]. The development of advanced delivery systems that improve the biological half-life of BH4 and its bioavailability in the CNS emerges as a strategy to enhance the efficacy of the current replacement therapy.

There exists a broad spectrum of synthetic biodegradable polymers used for the production of nanoparticulate drug delivery systems that improve the physicochemical stability and sustain the release of hydrophobic cargos [7]. Block copolymers made of hydrophilic components such as poly(ethylene glycol) (PEG) and hydrophobic polyester blocks such as poly(lactic acid), poly(lactic-*co*-glycolic acid), and poly(epsilon-caprolactone) (PCL) has gained major attention owing to the ability to fine-tune the hydrophilicity/lipophilicity and the thermal properties of the product and to consequently control the biodegradation and the release of the cargo in the biological environment [8,9,10,11,12,13,14,15]. However, BH4 is highly soluble (*S_0_* = 23 mg ml^−1^) and extremely chemically instable in water and air (Figure S1), precluding its encapsulation within polymeric NPs by any of the conventional methods.

Sepiapterin (SP, Figure 1b) is the natural precursor of BH4 and it is intracellularly converted into BH4 [16]. SP displays lower aqueous solubility (1.7 mg ml^−1^) and higher chemical stability than BH4, and it appears as a good candidate to replace it in the design of advanced drug delivery systems. At the same time, encapsulation of relatively hydrophilic molecules within hydrophobic polymeric NPs using conventional and eventually scalable preparation methods remains a challenge and the development of new encapsulation procedures is called for [17,18]. In addition, the release of hydrophilic small molecules from inherently hydrophobic nanocarriers is often uncontrolled and fast [19,20].

Hydrophilic (water-soluble) cyclodextrins (CDs) have been effectively applied in pharmaceutical development to increase the aqueous solubility and the physicochemical stability of hydrophobic drugs [21,22,23,24]. Much lesser works assessed the potential of hydrophobically-modified CDs to reduce the aqueous solubility of relatively hydrophilic compounds. Recently, peracylated CDs that are freely soluble in organic solvents such as ethanol and acetone and poorly water-soluble were proposed as excipients to decrease the water-solubility, prolong the biological half-life, and sustain the release of hydrophilic drugs through the formation of water-insoluble drug/CD complexes [25,26,27,28,29,30]. For example, 2,3,6-triacetyl-β-cyclodextrin (TAβCD) was proved to be an effective sustained release carrier for several hydrophilic drugs such as metformin, nicardipine, and flufenamic acid [24,25,26,27,28,29,30,31].

Aiming to encapsulate the relatively hydrophilic SP molecule within the hydrophobic domains of methoxy-poly(ethylene-glycol)-poly(epsilon-caprolactone) (mPEG-PCL) NPs and sustain its release, in this work, we explored for the first time the hydrophobization of SP with TAβCD as a preamble to the nano-encapsulation stage. For this, we studied two methods: (i) Drying with copolymer (DWC) that comprised the co-dissolution of SP, TAβCD, and mPEG-PCL in a mixture of acetone and methanol, drying under vacuum, re-dissolution, and nano-precipitation in water and (ii) preparation of an SP/TAβCD complex by the spray-drying of binary solutions using the Nano Spray Dryer B-90 HP and the later co-dissolution of the complex with the copolymer and their nano-precipitation in water. Dry powders were characterized by differential scanning calorimetry (DSC), Fourier-transform infrared spectroscopy (FTIR), and transmission and scanning electron microscopy (SEM and TEM, respectively), and compared to the pristine components of the complex and SP/TAβCD physical mixtures (PMs). Finally, the optimized complex was encapsulated within mPEG-PCL NPs by a direct nano-precipitation method. Overall results confirmed the promise of this simple and scalable strategy for the nano-encapsulation of SP.

## 2. Results and Discussion

### 2.1. BH4 and SP Stability

BH4 is known to undergo rapid degradation, while SP is known to be less sensitive to oxygen than BH4. The stability of BH4 and SP in deionized oxygen-free water (1% *w*/*v*) was investigated by UV/Vis spectrophotometry (Figure S1). The absorbance peak of BH4 at 266 nm decreased by 15% and red-shifted after 48 h, while a new absorbance peak at 329 nm already appeared after 1 h (Figure S1a). This also resulted in a change of color to yellow. Conversely, SP remained stable even after 8 days, confirming that this precursor was much more stable than BH4 and a better candidate for encapsulation (Figure S1b). Moreover, the intrinsic solubility of BH4 in water was higher than of SP and, thus, its encapsulation in hydrophobic polymers precluded. Thus, SP was chosen for further experiments.

For improvement of SP encapsulation within mPEG-PCL NPs, we assessed two strategies: (i) Drying of a solution of SP, TAβCD, and mPEG-PCL copolymer in acetone/methanol, re-dissolution in acetone and nano-precipitation in water, a method called DWC and (ii) spray-drying of a solution of SP and TAβCD in ethanol to produce the SP/TAβCD complex, and the subsequent dissolution of the complex and mPEG-PCL in acetone and nano-precipitation in water to obtain the SP-loaded NPs. In both cases, the nano-precipitation was performed according to the protocol described in the experimental section. Spray-dried SP/TAβCD complexes were fully characterized to get an insight on interactions occurring between the two components.

### 2.2. Characterization of Spray-Dried SP/TAβCD Complexes

In order to investigate possible SP/TAβCD interactions and exclude artifacts resulting from the sample preparation, pristine TAβCD (Figure 1c) was subjected to the same procedure (dissolution and spray-drying) as binary SP/TAβCD solutions; this sample was named as processed TAβCD. Pristine SP is extremely expensive and the Nano Spray Dryer B-90 HP requires relatively large amounts for sample collection. Thus, pristine SP was dissolved in ethanol, dried under vacuum, and used as the reference sample (processed SP). Two SP:TAβCD molar ratios were used for the preparation of complexes: (i) SP:TAβCD 1:1 and (ii) SP:TAβCD 1:2 (Table 1). PMs of the pristine components with the same molar ratio were prepared by grinding of dry TAβCD and SP using mortar and pestle and also used for comparison.

#### 2.2.1. Fourier-Transform Infrared Spectroscopy

**Figure 2:**
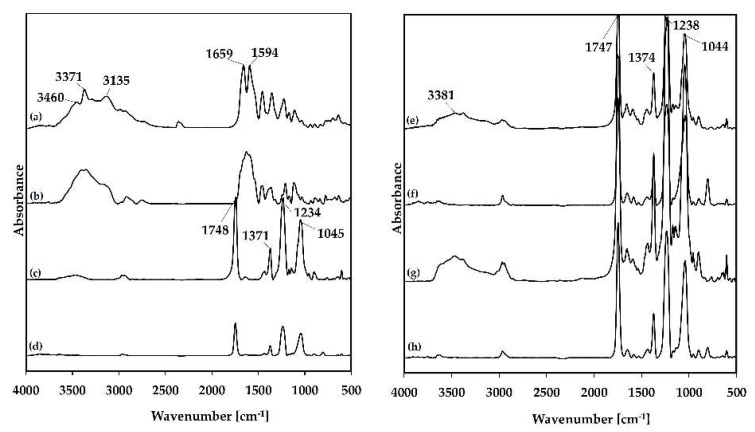
FTIR spectra of (**a**) pristine SP, (**b**) processed SP, (**c**) pristine TAβCD, (**d**) processed TAβCD, (**e**) SP1/TAβCD1 PM, (**f**) SP1/TAβCD1 SD, (**g**) SP1/TAβCD2 PM, (**h**) SP1/TAβCD2 SD.

FTIR spectra of SP and TAβCD (pristine and processed), their PMs and spray-dried SP/TAβCD complexes in the two molar ratios are shown in Figure 2. Pristine and processed SP showed a strong band at a region of 3800–2800 cm^−1^ (Figure 2a,b). Peaks at 3460 and 3371 cm^−1^ corresponded to N–H stretching vibration of primary amine and –OH stretching, a band at 3135 cm^−1^ due to the N–H stretching of secondary amine. Characteristic bands at 1659 and 1594 cm^−1^ were assigned to stretching vibrations of C=O and N-H bending of primary amine, respectively (Figure 2a,b). In addition, bands at 1458 and 1354 cm^−1^ corresponded to -CH_2_ asymmetric and symmetric bending, respectively, and at 1225 and 1111 cm^−1^ to stretching vibrations of C-O.

Pristine and processed TAβCD displayed very strong bands at 1748, 1371, 1234, and 1045 cm^−1^ that belong to C=O, and -CH_3_ and C-O-C vibrations of the acetyl group, respectively (Figure 2c,d) [31]. Spectra of PMs showed the overlapping of the bands of pristine SP and TAβCD and no significant shifts or depletion of the intensity of the characteristic bands with respect to the pristine components (Figure 2e,g). In contrast, FTIR spectra of the spray-dried SP/TAβCD mixture revealed a strong reduction or the complete disappearance of characteristic SP bands in the 3800–2800 cm^−1^ region, suggesting its strong interaction with TAβCD and the formation of a complex (Figure 2f,h) [21,22,23,24]. The inclusion or non-inclusion nature of this complex is difficult to discern. Bands of TAβCD were still apparent at 1747, 1374, 1238, and 1044 cm^−1^.

#### 2.2.2. Differential Scanning Calorimetry (DSC)

**Figure 3:**
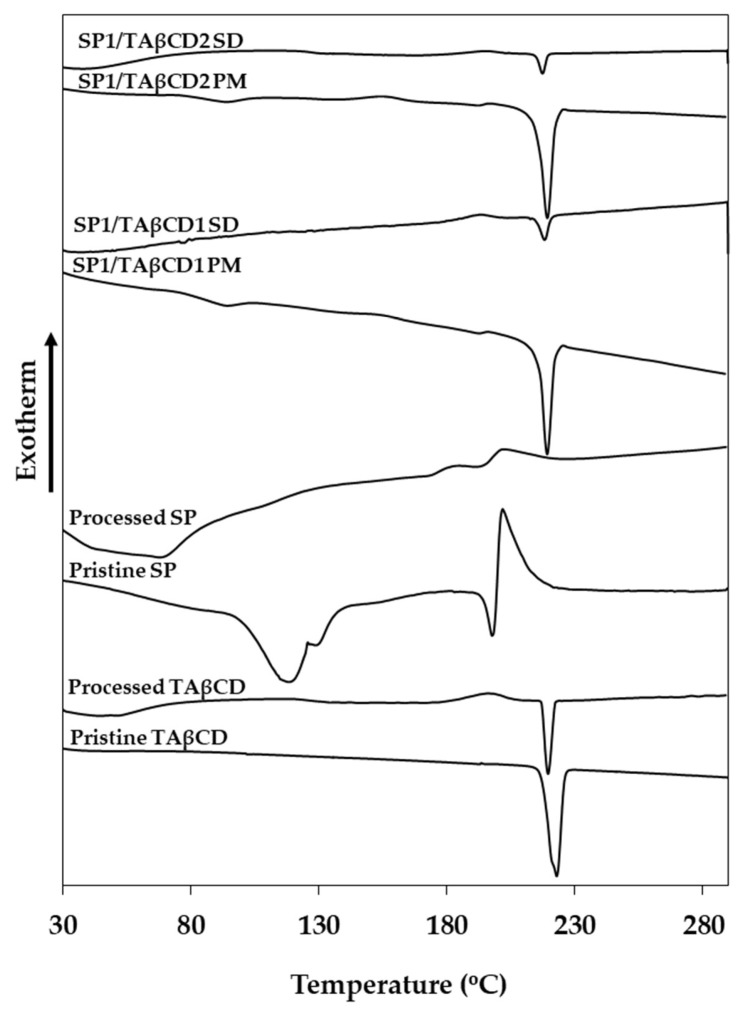
DSC thermograms of pristine and processed SP and TAβCD, their (physical mixtures (PMs) and the complexes obtained by spray-drying.

DSC is widely used to study the interaction between a drug and a CD in the solid state [21,32,33,34,35]. To confirm the formation of a complex, we compared the thermal behavior of pristine and processed SP and TAβCD, SP/TAβCD PMs and spray-dried SP/TAβCD by DSC. Pristine TAβCD was characterized by a sharp melting endotherm at 223 °C (Figure 3) associated with a melting enthalpy (ΔH_m_) of 43 J g^−1^ (Table 2). The thermal behavior of processed TAβCD differed from the one of the pristine counterparts; spray-dried TAβCD displayed an exothermic peak upon heating at 195 °C due to the crystallization of amorphous TAβCD with a crystallization enthalpy (ΔH_c_) of 8.4 J g^−1^ (Figure 3); the recrystallization of acetylated CDs that undergo amorphization during spray-drying was described elsewhere [34]. Then, recrystallized TAβCD melted at 220 °C, though a smaller ΔH_m_ of 13 J g^−1^ than in pristine TAβCD (43 J g^−1^), was observed (Table 2). This kind of behavior was also reported for TAβCD recrystallized from water/organic solvent solutions and indicated the partial amorphization of the CD during the processing [34]. Pristine SP showed a more complex thermal behavior. A broad endotherm at 116 °C (ΔH_m_ = 77.3 J g^−1^) that probably stemmed from the release of bound water (Figure 3, Table 2).

Then, the melting endotherm was observed at 197 °C (ΔH_m_ = 10 J g^−1^) followed by an exotherm at 201 °C of a larger relative area (ΔH = 48 J g^−1^). This exotherm could be associated with different thermal phenomena. On one hand, SP undergoes thermal decomposition upon heating. After analysis up to 280 °C, samples were recovered and visually inspected, showing a black color that suggested degradation. On the other hand, this exotherm could stem from the crystallization of metastable crystals via the liquid state. In this case, some metastable crystals that remained in the sample during melting served as nuclei for the recrystallization of the liquid phase upon heating. The existence of a broad spectrum of SP polymorphs has been recently published in a patent application though the only method used for their characterization was X-rays diffraction [36]. Processed SP showed a similar profile, though the water-related peak of pristine SP was not apparent in the processed counterpart, suggesting the efficient elimination of water residues available in the original sample by the deep vacuum drying (Figure 3, Table 2); the broad endotherm at 69 °C probably corresponded to the evaporation of organic solvent residues. Then, a weak endotherm at 171 °C could be assigned to the melting of semi-crystalline SP due to partial amorphization during the drying process, followed by a very weak exotherm. The fact that the energy associated with this second transition was substantially smaller for processed than for pristine SP indicated that it stemmed mainly from recrystallization and not from degradation. Otherwise, the ΔH of this exotherm in pristine and processed SP would have been similar, which was not the case.

The thermal analysis of PMs presented the endotherm associated with water release and the characteristic transitions of pristine SP and TAβCD. TAβCD crystallization during heating at lower temperatures resulted from recrystallization of an amorphous form obtained during the grinding in the preparation of the PM [29]. In addition, no significant reduction in the ΔH_m_ of TAβCD was observed; values being 40 and 43 J g^−1^ for SP1/TAβCD1 and SP1/TAβCD2 PMs, respectively. DSC analysis of spray-dried SP/TAβCD complexes showed the complete disappearance of the SP melting peak and a strong reduction in the ΔH_m_ of TAβCD and indicated the total SP and the partial TAβCD amorphization, and the formation of a SP/TAβCD complex. Considering that SP is a relatively hydrophilic molecule and that the cavity of TAβCD (as all the CDs) is more hydrophobic, the formation of inclusion complexes seems unlikely. Conversely, SP displays several functional groups that could undergo H bonding with the carbonyl groups of acetylated CD and, thus, contribute to the formation of non-inclusion complexes. Further studies are required to reveal the type of formed complex between this CD and SP. 

#### 2.2.3. Morphological Characterization of Spray-Dried Complexes

**Figure 4:**
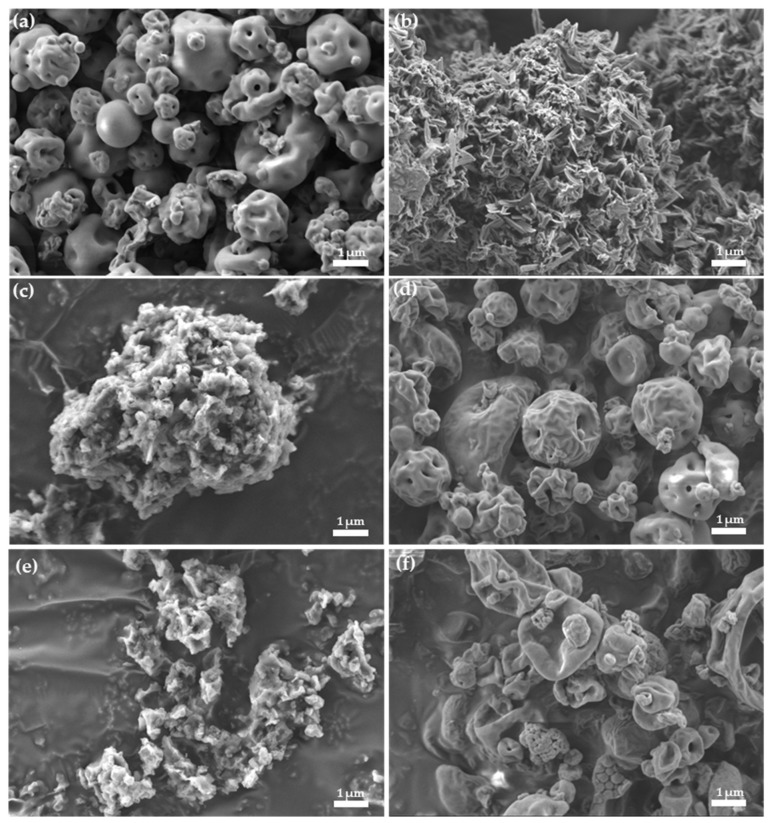
HR-SEM micrographs of (**a**) processed TAβCD, (**b**) processed SP, (**c**) SP1/TAβCD1 PM, (**d**) spray-dried SP1/TAβCD1 complex, (**e**) SP1/TAβCD2 PM, and (**f**) spray-dried SP1/TAβCD2 complex.

Morphological characterization of drug/CD complexes by electron microscopy is widely used to gain information about the nature of the components in the complex [33,35]. Nevertheless, even if an apparent difference in crystallization state of the raw materials compared to the products exists, this characterization method should be used to confirm the formation of a complex only when combined with other chemical and thermal characterization methods [37]. The surface aspect of processed SP and TAβCD, their PMs and the spray-dried complexes were visualized by HR-SEM (dry powders, Figure 4) and TEM (powders re-suspended in water and casted, Figure S2). In HR-SEM, processed (spray-dried) TAβCD appeared as round-shaped amorphous particles (0.5–5 µm) (Figure 4a). In addition, processed SP showed irregular elongated needle-like crystals formed due to its crystallization during solvent evaporation. HR-SEM micrographs of SP/TAβCD PMs revealed the presence of the SP crystals dispersed in TAβCD (Figure 4c,e) and suggested that regardless of the intimate mixing by grinding (a method used to produce drug/CD complexes), no molecular interactions between the two compounds were formed in solid state. Spray-dried mixtures appeared as round-shape particles, similar to spray-dried pristine TAβCD, with no visible SP crystals (Figure 4d,f). These results were consistent with DSC analysis and confirmed the amorphous nature of the spray-dried SP/TAβCD binary systems and the formation of the complex.

In addition, in TEM, processed TAβCD appeared as particles of an irregular shape (Figure S2a), while a processed SP sample produced by direct drop casting showed a needle-like crystalline morphology (Figure S2b). These results were similar to those obtained in HR-SEM. Both SP/TAβCD PMs showed the presence of square-shaped glassy chip structures that are typical for TAβCD (Figure S2c,e), while these structures were not observed in spray-dried complexes (Figure S2d,f), as reported elsewhere [34]. These observations supported that both components undergo amorphization during spray-drying.

### 2.3. Production and Characterization of SP-Loaded NPs

#### 2.3.1. mPEG-PCL Copolymer Synthesis

A mPEG-PCL copolymer with a relatively low hydrophilic-lipophilic balance was chosen as a model for NP production. The copolymer was synthesized by the ring opening polymerization (ROP) of epsilon-caprolactone (CL) initiated by the terminal hydroxyl group of a methoxy-terminated PEG with a molecular weight of 4000 g mol^−1^ in the presence of tin(II) 2-ethylhexanoate (SnOct) as catalyst at 145 °C for 2.5 h and in the appropriate mPEG:CL molar ratio to obtain a PCL block of approximately 20,000 g mol^−1^ (Figure S3). The successful polymerization was confirmed by proton nuclear magnetic resonance spectroscopy (^1^H-NMR) (Figure S4) and the number average molecular weight (M_n_), the weight average molecular weight (M_w_), and the dispersity (Đ, M_w_/M_n_) of the copolymer measured by gel permeation chromatography (GPC) (Table S1). Figure S4 shows a representative ^1^H-NMR spectrum of the obtained mPEG-PCL copolymer. The peak at δ = 3.60 ppm was assigned to the methylene (–CH_2_) protons of the PEG chain. In addition, characteristic peaks of the methylene protons of the PCL block appeared at δ = 2.26, 1.61, 1.35, and 4.02 ppm. Since the number-average molecular weight of the PEG block used for the reaction was known from the supplier, the molecular weight of the PCL block was calculated by taking the integration ratio of the characteristic peak of PEG (δ = 3.60 ppm) and PCL (δ = 2.26 ppm) (Table S1), as described elsewhere [38]. The experimental M_n_ determined by ^1^H-NMR is 25,000 g/mol (Table S1). In addition, the molecular weight of the copolymer was measured by GPC (Table S1); M_n_ and M_w_ values being 19,000 and 32,000 g/mol, respectively, and the Đ 1.71.

#### 2.3.2. Nanoencapsulation of SP

**Figure 5:**
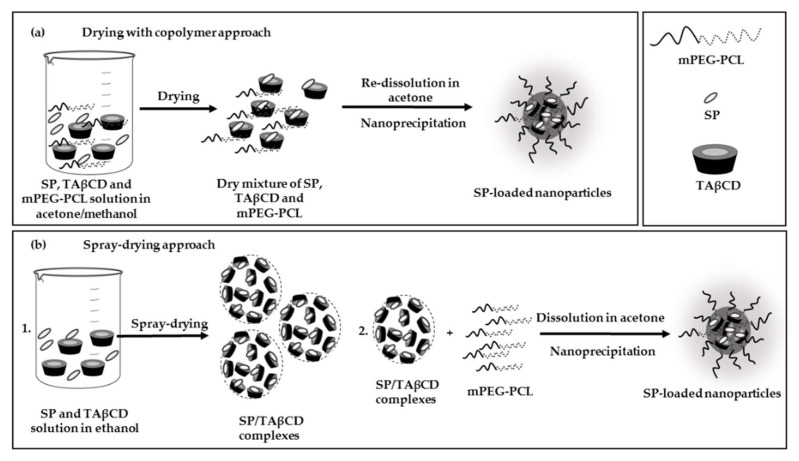
Methods for the encapsulation of SP. (**a**) Drying of a solution of SP, TAβCD, and mPEG-PCL copolymer prior to re-dissolution and nano-precipitation and (**b**) co-dissolution of spray-dried SP/TAβCD complex and mPEG-PCL copolymer and nano-precipitation.

Pristine SP was encapsulated within mPEG-PCL NPs by a modified nano-precipitation method performed in a flask under inert nitrogen conditions to prevent the possible oxidation of SP by ambient oxygen and protected from light because SP is light-sensitive. Aiming to improve the SP loading within the NPs, we assessed the encapsulation by using two methods (Figure 5).

The first method comprised the dissolution of SP, TAβCD, and mPEG-PCL in a mixture of acetone end methanol, drying, and re-dissolution in acetone with subsequent nano-precipitation in water for NP formation (Figure 5a). Conversely, in the second, a spray-dried SP/TAβCD complex was co-dissolved with the copolymer in acetone and this solution used for the nano-precipitation (Figure 5b). Equivalent amounts of each component were used in both cases, as depicted in Table S2. As for the first approach, we aimed to promote interactions between hydrophobic TAβCD and slightly more hydrophilic SP, as well as between hydrophobic PCL blocks of the copolymer and TAβCD and, by doing so, to increase the entrapment of SP molecules in the hydrophobic PCL/TAβCD matrix formed during the nano-precipitation process with respect to TAβCD-free counterparts.

#### 2.3.3. Size and Size Distribution of SP-Loaded NPs

The size (hydrodynamic diameter, D*_h_*, estimated as intensity size distribution and Z-average), the broadness of each size population (expressed as standard deviation from the size mean in nm) and the width of the size distribution calculated from the cumulant analysis (polydispersity index, PdI) of blank (made only with the copolymer) and SP-loaded NPs produced by both methods, as well as suspensions of pristine and processed TAβCD produced under the same conditions were measured by dynamic light scattering (DLS) (Table 3). Initially, we characterized the size of nano-precipitated TAβCD that showed a size of ˃200 nm that is consistent with the formation of CD aggregates in water, even at relatively low concentrations [39]. The aggregation trend increased for this hydrophobized CD that displayed poor aqueous solubility. Blank NPs made of mPEG-PCL without SP and CD showed a small D*_h_* of 65 nm and an S.D. (expression of the peak width) of 28 nm. When SP was nano-encapsulated directly by nano-precipitation without the incorporation of TAβCD, the size gradually increased to 73 nm (S.D. = 26 nm) for 1 mg and 83 nm (S.D. = 29 nm) for 2 mg of SP (Table 3). However, most SP remained outside of the nanoparticle (see below). All the SP-loaded NPs produced by using the DWC method, with the exception of the SP1/TAβCD2 formulation that used 1 mg of SP, showed two size populations: One major in the nanoscale (72–100 nm) and one minor in the microscale (4.2–5 µm) (Table 3). SP1/TAβCD2 DWC with 1 mg SP resulted in NPs with monomodal size distribution, D*_h_* of 105 nm and S.D. of 30 nm. These results could be explained by the poor mixing between both hydrophobic components, TAβCD and PCL, in the NP. This method resulted in very low encapsulation, as described in the following section. Conversely, a spray-dried complex containing the optimized ratio between SP and the CD, namely SP1/TAβCD1, formed NPs of small D*_h_* (74–75 nm) and smaller S.D. (21 to 22 nm) than the rest of formulations (Table 3). The fact that NPs produced by using a higher TAβCD content (SP1/TAβCD2) displayed D*_h_* of 69–72 nm accompanied by much larger structures strongly suggests that part of the CD could not be incorporated into the NP matrix and formed typical CD aggregates of several hundreds of nanometers and up to micron-size structures [39].

#### 2.3.4. Encapsulation Efficiency and SP Loading

Two parameters, the encapsulation efficiency (%*EE*) and the drug loading (%*DL*), of SP in mPEG-PCL NPs, were quantified indirectly, as reported elsewhere for other drugs [40]. For this, SP-loaded NP suspensions were washed thoroughly to remove residues of free TAβCD and SP, and free SP quantified in the filtrate fraction and the *%EE* calculated according to Equation (1)

(1) $$\%\mathrm{EE}\;=\;\frac{{\mathrm{SP}}_{\mathrm{NP}}}{\mathrm{SPt}}\;\times \;100\%$$

where SP_NP_ is the weight of SP in the NPs and SP_t_ is the total weight of SP used in the encapsulation process.

In addition, the *%DL* was calculated according to Equation (2)

(2) $$\%\mathrm{DL}\;=\;\frac{{\mathrm{SP}}_{\mathrm{NP}}}{\mathrm{NPt}\;}\;\times \;100\%$$

where SP_NP_ is the weight of SP in the NPs and NP_t_ is the total weight of NPs used for the quantification.

SP is a relatively hydrophilic molecule and, thus, its water-soluble nature makes it difficult for physical loading within hydrophobic mPEG-PCL NPs without the incorporation of the hydrophobic CD, resulting in %*EE* and %*DL* of 9–14% and 0.2–0.6%, respectively (Table 3). Similar or even lower values were obtained with DWC systems that failed to improve the encapsulation. These results were consistent with DLS data, confirming that, regardless of the presence of TAβCD, in this method, there was no effective entrapment of SP molecules within the PCL domains of the NP. When the CD was used to hydrophobize SP by spray-drying, both %*EE* and %*DL* increased, the highest values (85% and 2.6%, respectively) being obtained for SP-loaded NPs produced with SP1/TAβCD1 SD and 2 mg of SP in the nano-precipitation. Investigating the shelf-life of the nano-formulations was beyond the scope of this work. At the same time, it is important to mention that storage in darkness and at 4 °C did not prevent the apparent degradation of SP visualized with the naked eye by a clear color loss of the yellow SP. In the future, efforts will need to be devoted to finding optimal storage conditions to ensure longer physicochemical stability that is compatible with the bench-to-bedside translation. 

#### 2.3.5. Morphological Characterization of SP-Loaded NPs

**Figure 6:**
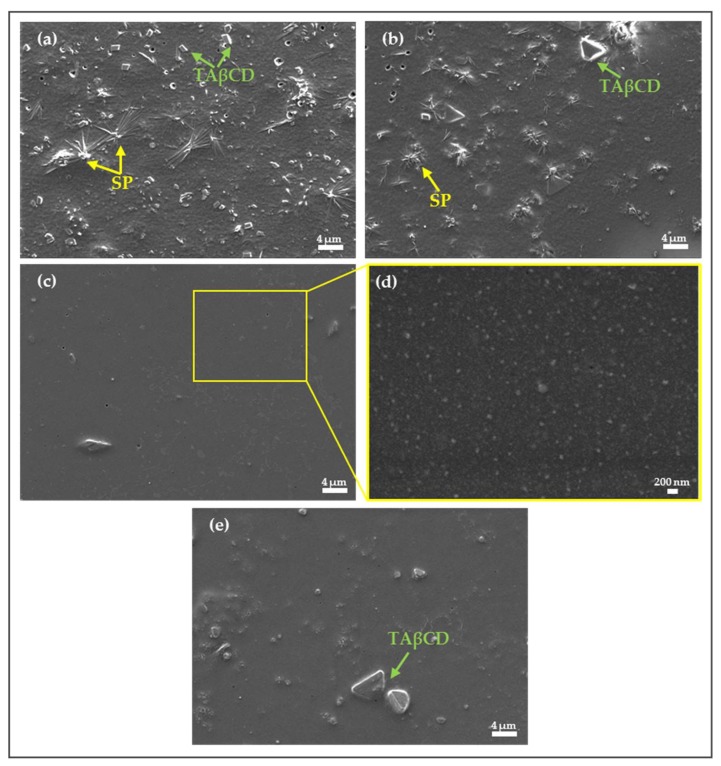
HR-SEM micrographs of SP-loaded mPEG-PCL NPs by using (**a**) SP1/TAβCD1 DWC (1 mg of SP), (**b**) SP1/TAβCD1 DWC (2 mg of SP), (**c**) encapsulation of spray-dried SP1/TAβCD1 complex (1 mg of SP) under ×5K, (**d**) ×50K magnification of (**c**) and (**e**), and encapsulation of spray-dried SP1/TAβCD2 complex (1 mg of SP).

Representative SP-loaded NPs were visualized by using HR-SEM (Figure 6). For this, NP suspensions were drop-casted on a silicon wafer. Upon water evaporation and drying of the sample, free SP undergoes crystallization and forms needle-like crystals, similar to those observed during TEM analysis (Figure 6b). Free TAβCD can also undergo crystallization upon drying and form well- defined prismatic crystals or, conversely, to remain amorphous and form glassy chips [34]. As it can be seen, in SP-loaded NPs prepared by the DWC method using 1 and 2 mg equivalent amounts of SP (Figure 6a,b, respectively), both SP and TAβCD crystals were observed, confirming the presence of free SP and TAβCD in the NP suspension. As for SP-loaded NPs prepared using spray-dried complexes, in the case of SP1/TAβCD1 SD with 1 mg of SP, SP or TAβCD crystals were not visible (Figure 6c,d). However, in SP1/TAβCD2 SD produced with 1 mg of SP, several glassy TAβCD chips were visualized. These findings indicated that a higher relative weight fraction of TAβCD used in the production of the complex resulted in an excess of TAβCD, which was not efficiently entrapped within the PCL matrix of the NP formed during the nano-precipitation. In other words, the excess of TAβCD precluded the formation of stable mPEG-PCL NPs. Overall these observations were in a good agreement with DLS data and confirmed that additional size populations observed in DLS analysis were associated with the presence of free or aggregated TAβCD.

#### 2.3.6. SP Release Study

**Figure 7:**
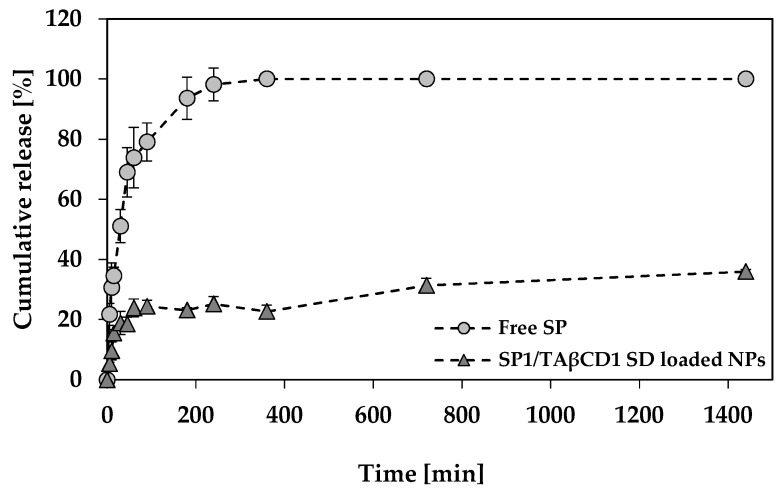
Cumulative release of free SP and SP from SP1/TAβCD encapsulated PEG-PCL NPs, over 24 h.

SP-loaded NPs with the highest *%EE* and *%DL* obtained by using SP1/TAβCD1 SD complex (2 mg SP equivalent) were used for the evaluation of the SP release using the dialysis membrane method. The release of SP from NPs was monitored as a function of time and compared to the profile of free SP. The concentration of free SP solution was equal to the concentration of SP in the NP system assuming 100% release from the NPs (calculated based on *%DL* of the SP-loaded NPs). The amount of released SP expressed as the cumulative percentage *versus* time was plotted and then, release data fitted to different release models by using the DDSolver software [41]. Cumulative SP release profiles are shown in Figure 7. As expected, free SP was released from the dialysis bag very fast (almost 100% within 150 min). These results indicated that the dialysis bag did not substantially hinder the release, as opposed to larger molecules such as rifampicin that were retained longer [42]. A marked reduction in the SP release rate was observed for the NPs produced using the SP1/TAβCD1 SD complex with only 40% released after 24 h. These results are remarkable for a molecule that displays relatively good water solubility. The release of free SP fitted well first order kinetics (*k_1_* = 0.029 min^−1^; R^2^ = 0.939) (Figure S5a), this sustained release effect being exclusively ascribed to the dialysis membrane. Conversely, the release of SP from the NPs fitted the Korsmeyer−Peppas model (*k_KP_* = 8.21 min^−1^; R^2^ = 0.933) (Figure S5b) with *n* = 0.204 that, considering that the release system is spherical, indicated a mainly diffusive release mechanism from more polydisperse systems such as our NPs that are not monodisperse in size. These results are in good agreement with the general observations throughout the characterization of the spray-dried SP/TAβCD complexes that TAβCD efficiently hydrophobizes SP and improves its entrapment within the hydrophobic PCL domain of the NP.

## 3. Materials and Methods

### 3.1. Preparation of Spray-Dried SP/TAβCD Complexes

TAβCD (85 or 170 mg for 1:1 and 1:2 SP/TAβCD complexes, respectively; Sigma-Aldrich, St. Louis, MO, USA;) was dissolved in ethanol (19 and 38 mL for 1:1 and 1:2 complexes, respectively; Gadot, Netanya, Israel) with assistance of sonication in an ultrasonic bath (5 min, Elmasonic S 30, Elma Schmidbauer GmbH, Singen, Germany). SP (10 mg; Schricks Laboratories, Bauma, Switzerland) was dissolved in ethanol (12 mL) and mixed with the TAβCD ethanol solution. The resulting binary solution was stirred under heating at 55 °C (10 min; Hei-Tec Magnetic Stirrer, Heidolph Instruments, Schwabach, Germany) in order to prevent TAβCD precipitation and subsequently spray-dried (Nano Spray Dryer B-90, Büchi Labortechnik AG, Flawil, Switzerland) using a closed loop configuration, under the following conditions: Nitrogen flow 20 mL min^−1^, an inlet temperature of 55 °C, an outlet temperature of 60 °C and 80% spraying. The obtained powder was kept in a sealed vial at 4 °C and protected from light until use.

Pristine TAβCD was spray-dried using the same method and named as processed TAβCD. An SP reference sample (processed SP) was prepared by dissolution of SP (10 mg) in ethanol (12 mL) and drying under vacuum (Vacuum Oven Lab-Line Instruments Inc., Dubuque, IL, US).

### 3.2. Preparation of PMs

PMs of SP and TAβCD were prepared by mixing the pristine substances (1 mg of SP with 8.5 or 17 mg of TAβCD for 1:1 and 1:2 SP/TAβCD PM, respectively) using a geometric dilution method by continually grinding substances in a mortar and pestle.

### 3.3. Characterization of Spray-Dried SP/TAβCD Complexes

Spray-dried SP/TAβCD complexes (SD SP/TAβCD) were fully characterized with DSC, FT-IR, SEM, and TEM in order to confirm the formation of the complex and not of a PM.

#### 3.3.1. Differential scanning colorimetry

DSC analysis was performed in a DSC 2 STAR^e^ system simultaneous thermal analyzer with STAR^e^ software V13 (Mettler-Toledo, Schwerzenbach, Switzerland) at a heating rate of 10 °C min^−1^ in the 25 to 300 °C temperature range under a nitrogen flow of 20 mL min^−1^ and using In as standard.

#### 3.3.2. Fourier-Transform Infrared Spectroscopy

FTIR was recorded in an Equinox 55 spectrometer (Bruker Optics Inc., Ettlingen, Germany). Each sample (0.3% *w*/*w*) was thoroughly grinded with powdered KBr (Merck Chemical GmbH, Darmstadt, Germany) and compressed to a pellet under pressure of 10 MPa before the analysis. Spectra were obtained in the wavenumber range of 4000–500 cm^−1^ with a resolution of 4 cm^−1^ and 32 scans were performed for each spectrum.

#### 3.3.3. Scanning Electron Microscopy

The surface morphology of the pristine components and their binary combinations was visualized by HR-SEM (Zeiss Ultra-Plus FEG-SEM, Zeiss, Berkin, Germany), equipped with a high-resolution field emission gun. Samples were carbon sputtered prior to observation. The acceleration voltage was 2–4 kV. Images were obtained using a secondary electron detector at 3–4 mm working distance.

#### 3.3.4. Transmission Electron Microscopy

TEM was carried out in a Technai G2 T20 S-Twin (FEI, Eindhoven, Netherlands), operated at 200 kV. Samples were dissolved in water, followed by placing three 10 µL drops one after the other on a carbon grid (Formvar/Carbon 300 mesh; Electron Microscopy Sciences, Hatfield, PA, USA). Samples were finally dried in a fume hood overnight before analysis.

### 3.4. Preparation of SP-Loaded mPEG-PCL NPs

#### 3.4.1. Synthesis of mPEG-PCL Copolymer

An mPEG-PCL block copolymer was synthesized by a solvent-free melt ROP of CL (5 g; Sigma-Aldrich) initiated by the terminal hydroxyl group of mPEG of molecular weight 4000 g mol^−1^ (0.5 g; Tokyo Chemical Industry Co. Ltd. Tokyo, Japan). The polymerization was catalyzed by SnOct (142 µL, 1:200 molar ratio to CL, Sigma-Aldrich) and carried out at 145 °C (2.5 h) under nitrogen atmosphere (Figure S3). After the reaction, the crude mixture was cooled down to room temperature, dissolved in dichloromethane (Gadot), and precipitated in an excess of diethyl ether (Bio-Lab Ltd., Jerusalem, Israel). The precipitated mPEG-PCL copolymer was filtered to remove remaining unreacted reagents, washed several times with diethyl ether, vacuum-dried at room temperature until constant weight, and stored at −24 °C until use. The formation of the copolymer was determined by ^1^H-NMR at 400 MHz by using a Bruker Avance III High Resolution spectrometer (Bruker BioSpin GmbH, Rheinstetten, Germany). The M_n_, M_w_, and Đ (M_w_/M_n_) of the copolymer were determined by GPC (Alliance HPLC System, Waters Corp., Milford, MA, USA) with refractive index detector and 4 Styragel^®^ HR (1–4) columns (7.8 × 300 mm, packed with 5 µm particles, Waters Corp.). The sample was prepared by dissolving mPEG-PCL copolymer (1% *w*/*v*) in tetrahydrofuran (THF, HPLC grade, Bio-Lab) and injecting 20 μL, and the runs were conducted with a mobile phase flow of 1 mL min^−1^, at 40 °C. Poly(methyl methacrylate) standards (PSS polymer standards service, Mainz, Germany) with molecular weights between 2260–171,000 g mol^−1^ were used for molecular weights calibration.

#### 3.4.2. Drying of SP and TAβCD with mPEG-PCL Copolymer

In the dissolution with copolymer (DWC) method, SP, TAβCD, and mPEG-PCL (equivalent amounts of each component are detailed in Table S2) were dissolved in an acetone:methanol mixture (1:5 volume ratio) and magnetically stirred for 1 h at room temperature. Then, solvents were evaporated in a rotary evaporator (Rotavapor^®^ R-100, Büchi Labortechnik AG) at room temperature and the dry solid mixture of SP, TAβCD, and mPEG-PCL was re-dissolved in anhydrous acetone. Nano-precipitation was performed as described below.

#### 3.4.3. Encapsulation of SP within mPEG-PCL NPs

The encapsulation of pristine SP and SD SP/TAβCD complexes in mPEG-PCL NPs was performed using the nano-precipitation method. In brief, the mPEG-PCL copolymer and SD SP/TAβCD complex (or pristine SP) were dissolved in anhydrous acetone (10 mL) and the tertiary copolymer solution was added dropwise to the degassed deionized distilled water (50 mL) in a sealed round-bottom flask under nitrogen flow to prevent the oxidation of SP due to exposure to air using a syringe pump (Laboratory Syringe Pump SYP-01, MRC Laboratory Equipment Manufacturer, Kfar Saba, Israel) at an injection rate of 0.333 mL min^−1^ and under magnetic stirring (480 rpm, Hei-Tec Magnetic Stirrer). Equivalent amounts of each component used in this method are indicated in Table S2. Then, the acetone was evaporated using a rotary evaporator (Rotavapor^®^ R-100) at room temperature. The NP suspension was kept in a sealed vial at 4 °C and protected from light until use. The production of blank mPEG-PCL NPs (SP- and TAβCD-free) was carried out using the same method, though without the addition of SP and TAβCD. SP was also directly nano-encapsulated within mPEG-PCL NPs without CD by nano-precipitation. This sample was named Pristine SP NPs. Since CDs aggregate in water [38], we also characterized TAβCD that was spray-dried, re-dissolved in acetone, and nano-precipitated in water, exactly the same way as for the production of the NPs using the spray-dried complex, though without the addition of SP and the copolymer.

### 3.5. Characterization of SP-Loaded NPs

#### 3.5.1. Size and Size Distribution

*D_h_* (estimated by intensity size distribution and Z-average), the S.D. of each size population (a measure of the size population width; expressed in nm) and the PdI of the different NPs were measured by means of DLS (Zetasizer Nanoseries ZS90, Malvern Instruments, Malvern, UK). Samples were pre-tested using different concentrations to minimize particle–particle interactions and multiple light scattering that may lead to sizes that are larger than the actual ones, as recommended in the ISO 22412:2017 [43]. Results are presented as a mean value of three independent measurements and each analysis is the result of at least five runs.

#### 3.5.2. SP Encapsulation Efficiency and Drug Loading

For quantification of %*EE* and *%DL* and free SP was separated from the NPs by ultrafiltration in Amicon^®^ Ultra 15 mL Filters (MWCO 100 kDa, Merck Chemicals GmbH) that were washed according to the manufacturer instructions before use to remove any residues that might interfere with the SP quantification. For this, each sample was centrifuged at 4500×*g* for 15 min at room temperature, and SP was quantified in the filtrate fraction in a plate reader (Multiskan GO Microplate Spectrophotometer with SkanIt^TM^ software, Thermo Fisher Scientific Oy, Vantaa, Finland) employing a calibration curve of SP in water at 420 nm built in a range between 10 and 100 µg ml^−1^ (R^2^ = 0.996). All experiments were performed in triplicate.

#### 3.5.3. Morphological Analysis of SP-Loaded NPs

Representative samples of SP-loaded NPs were visualized by HR-SEM (Zeiss Ultra-Plus FEG-SEM). Samples were prepared by drop casting of 0.1% *w*/*v* NP suspension on a silicon wafer (CZ polished silicon wafers; SEH Europe Ltd., West Lothian, U.K.). Samples were carbon sputtered prior to analysis. The acceleration voltage was 2 to 4 kV. Images were obtained using a secondary electron detector at a 3 to 4 mm working distance.

#### 3.5.4. SP Release Study

SP release from the NPs was evaluated using the dialysis membrane diffusion technique utilizing phosphate-buffered saline (0.1 M PBS of pH 7.4) as release medium prepared with potassium phosphate dibasic (K_2_HPO_4_, Spectrum chemical MFG Corp., Gardena, CA, USA) and monobasic (KH_2_PO_4_, EMD Millipore corp., Billerica, MA, USA). Immediately after preparation, SP1/TAβCD1 SD loaded NPs (2 mg SP equivalent) were washed from free SP using Amicon^®^ as described above (Section 3.5.2) and 5 mL of washed NPs was placed in a dialysis membrane (regenerated cellulose tubular membrane, molecular weight cutoff of 3500 Da, Membrane Filtration Products, Inc., Seguin, TX, USA) and immersed in 0.5 L of release medium at 37 °C. Aliquots of release medium (3 × 1 mL) were removed at predetermined time intervals and replaced with fresh preheated (37 °C) PBS to keep the total volume constant. SP release from the NP formulation was compared to the release of free SP from dialysis membrane (34 µg mL^−1^ SP solution in water). The concentration of SP in the release medium at each time point was measured using the plate reader (at 420 nm, Multiskan GO Microplate Spectrophotometer with SkanItTM software) and a calibration curve of SP in PBS built in a range between 0.1 and 30 µg mL^−1^ (R^2^ = 1). Release assays were performed in triplicate and results are expressed as mean ± SD. Average release data was further fitted to different drug release models using DDSolver version 1.0, a free calculation program used to analyze dissolution or fit drug release data [41].

## 4. Conclusions

In the present work, we investigated for the first time a new strategy for the encapsulation of a relatively hydrophilic cargo, SP, in the hydrophobic domains of mPEG-PCL NPs. SP/TAβCD solid complexes were successfully prepared by spray-drying. Under the present conditions, the highest *%EE* and *%DL* were achieved with an SP/TAβCD SD complex at a 1:1 molar ratio. In vitro release studies revealed that the encapsulation of SP with the assistance of the complex sustained the release of the cargo as opposed to the free counterpart and of other hydrophilic drugs such as didanosine that were released from similar NPs very fast [19,20]. Overall, the results of this study indicate that SP/TAβCD spray-dried complexes can be used for the hydrophobization of SP and its further nano-encapsulation within hydrophobic polymeric NPs. Future work will be dedicated to improving both *%EE* and *%DL*, to find optimized storage conditions that prolong the shelf-life of the nano-formulations and to assess the effect of the nano-encapsulation on the pharmacokinetics.

## Figures and Tables

**Table 1:** Nomenclature of SP/TAβCD complexes and their physical mixtures (PMs).

Preparation Method	SP:TAβCD Molar Ratio	Nomenclature
Spray-drying (SD) ^a^	1:1	SP1/TAβCD1 SD
	1:2	SP1/TAβCD2 SD
Drying with copolymer (DWC) ^b^	1:1	SP1/TAβCD1 DWC
	1:2	SP1/TAβCD2 DWC
Physical mixture (PM) ^c^	1:1	SP1/TAβCD1 PM
	1:2	SP1/TAβCD2 PM

^a^ SP/TAβCD complex produced by spray-drying in a Nano Spray Dryer B-90 HP. ^b^ Co-dissolution of SP, TAβCD and mPEG-PCL in acetone/methanol and drying under vacuum. ^c^ Grinding of a mixture of SP and TAβCD with mortar and pestle.

**Table 2:** Thermal analysis of pristine and processed SP and TAβCD, spray-dried complexes and PMs, as determined by differential scanning calorimetry (DSC). Enthalpy values were normalized to TAβCD and SP content.

Sample	TAβCD				SP	
	T_m_ [°C]	ΔH_m_ [J g^−1^]	T_c_ [°C]	ΔH_c_ [J g^−1^]	T_m_ [°C]	ΔH_m_ [J g^−1^]
Pristine TAβCD	223	43	-	-	-	-
Processed TAβCD	220	13	195	8.4	-	-
Pristine SP	-	-	-	-	190	-
Processed SP	-	-	-	-	171	-
SP1/TAβCD1 SD	219	7.3	193	4.7	-	-
SP1/TAβCD1 PM	219	40	153	3.2	-	-
SP1/TAβCD2 SD	217	4.2	195	4.7	-	-
SP1/TAβCD2 PM	219	43	155	5.6	-	-

**Table 3:** Characterization of SP-loaded nanoparticles (NPs). Nano-precipitated TAβCD and blank NPs are included for comparison.

Sample	SP Equivalent Amount used for Encapsulation [mg]	D*_h_* [nm] ^a,b^ (± S.D.)	%Intensity ^b^	S.D. [nm] ^b,c^	Z-average [nm] ^b^ (± S.D.)	PdI ^b^	*%EE* [%] ^d^ (± S.D.)	*%DL* [%] ^d^ (± S.D.)
Processed TAβCD ^e^	-	312 (25)	100	66	278 (15)	0.23	-	-
Blank NPs ^f^	-	65 (3)	100	28	58 (2)	0.16	-	-
Pristine SP NPs ^g^	1	73 (3)	100	26	65 (1)	0.13	9 (1)	0.2 (0.1)
	2	83 (2)	100	29	74 (1)	0.10	14 (1)	0.6 (0.1)
SP1/TAβCD1 DWC NPs	1	72 (7) 353 (71)	84 16	13 62	156 (29)	0.36	0 (0)	0 (0)
	2	100 (25) 4260 (226)	86 14	41 369	183 (47)	0.42	11 (1)	0.4 (0.1)
SP1/TAβCD2 DWC NPs	1	105 (5)	100	30	109 (5)	0.27	7 (1)	0.1 (0)
	2	99 (14) 5066 (546)	77 23	15 560	69 (1)	0.75	9 (1)	0.3 (0)
SP1/TAβCD1 SD NPs	1	74 (1)	100	22	67 (1)	0.10	62 (1)	1.1 (0.1)
	2	75 (2)	100	21	69 (2)	0.10	85 (1)	2.6 (0.1)
SP1/TAβCD2 SD NPs	1	72 (1) 5105 (496)	89 11	14 593	134 (42)	0.39	50 (1)	1.5 (0.1)
	2	69 (3) 312 (35) 4071 (852)	51 40 9	22 43 731	260 (30)	0.69	53 (1)	0.9 (0.1)

^a^ D*_h_* are the intensity distribution values and expressed as the average of 3 independent experiments (*n* = 3) ± S.D. ^b^ Determined by dynamic light scattering (DLS). ^c^ Standard deviation (S.D.) of each size population that is an expression of the peak width. ^d^ Determined by UV-Vis spectrophotometry. ^e^ To produce processed TAβCD, the CD was spray-dried, re-dissolved in acetone, and nano-precipitated in water. ^f^ NPs made only of mPEG-PCL copolymer. ^g^ SP-loaded nanoparticles produced by the direct nano-encapsulation of SP without the incorporation of TAβCD.

## Supplementary Materials

The following are available online, Figure S1. UV-Vis spectra of (a) pristine BH4 and (b) pristine SP solutions in water (1% *w*/*v*) at different times. Figure S2. TEM micrographs of (a) processed TAβCD, (b) processed SP, (c) SP1/TAβCD1 PM, (d) spray-dried SP1/TAβCD1 complex, (e) SP1/TAβCD2 PM, and (f) spray-dried SP1//TAβCD2 complex. Figure S3. Ring opening polymerization of CL initiated by the hydroxyl groups of mPEG with a molecular weight of 4000 g mol^−1^. Figure S4. The ^1^H-NMR spectrum of the mPEG-PCL copolymer in CDCl_3_. Figure S5. Fitting of the average release data of (a) free SP to zero-order kinetics and (b) SP from SP1/TAβCD encapsulated PEG-PCL NPs to the Korsmeyer-Peppas model, as determined with DDSolver Software 1.0. Table S1. The number average molecular weight (M_n_), the weight average molecular weight (M_w_), and the dispersity (Đ, M_w_/M_n_), as determined by ^1^H-NMR and GPC. Table S2. Equivalent amounts of the different components used for the encapsulation of SP within mPEG-PCL NPs.
