# Supplementary material for: Enhanced Nanoencapsulation of Sepiapterin within PEG-PCL Nanoparticles by Complexation with Triacetyl-Beta Cyclodextrin
Source: Molecules. 2019 Jul 26;24(15):2715. doi: 10.3390/molecules24152715 (PMC6695667; doi:10.3390/molecules24152715)
Supplement: Supplementary file 1 [file molecules-24-02715-s001.pdf]

molecules
MDPI

Supplementary material

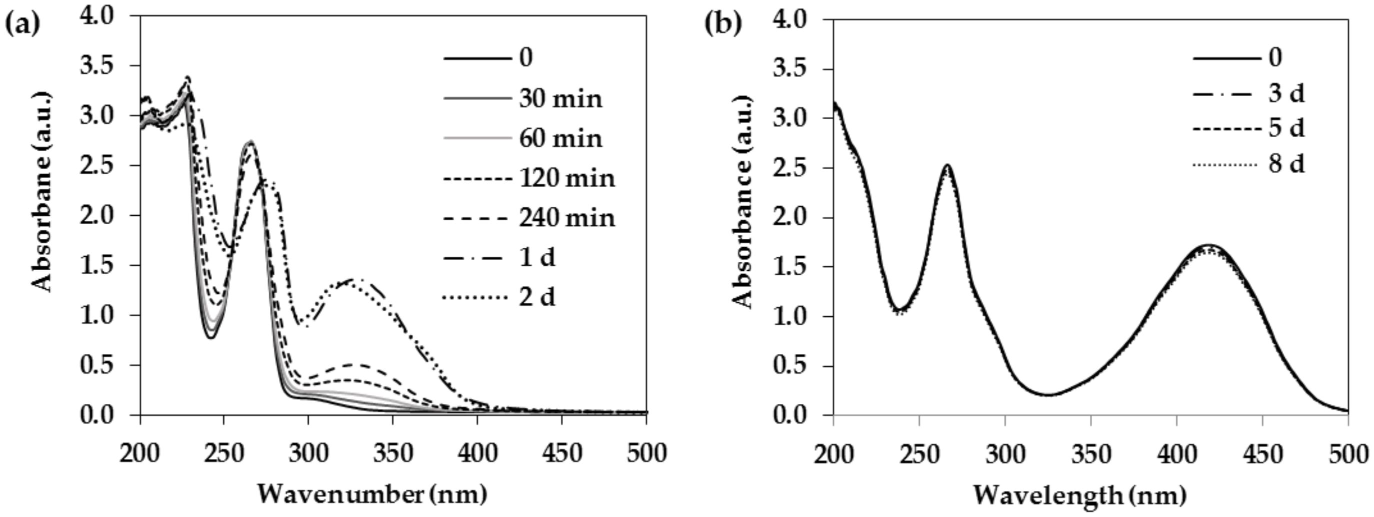

Figure S1. UV-Vis spectra of (a) pristine BH4 and (b) pristine SP solutions in water (1% w/v) at different times.

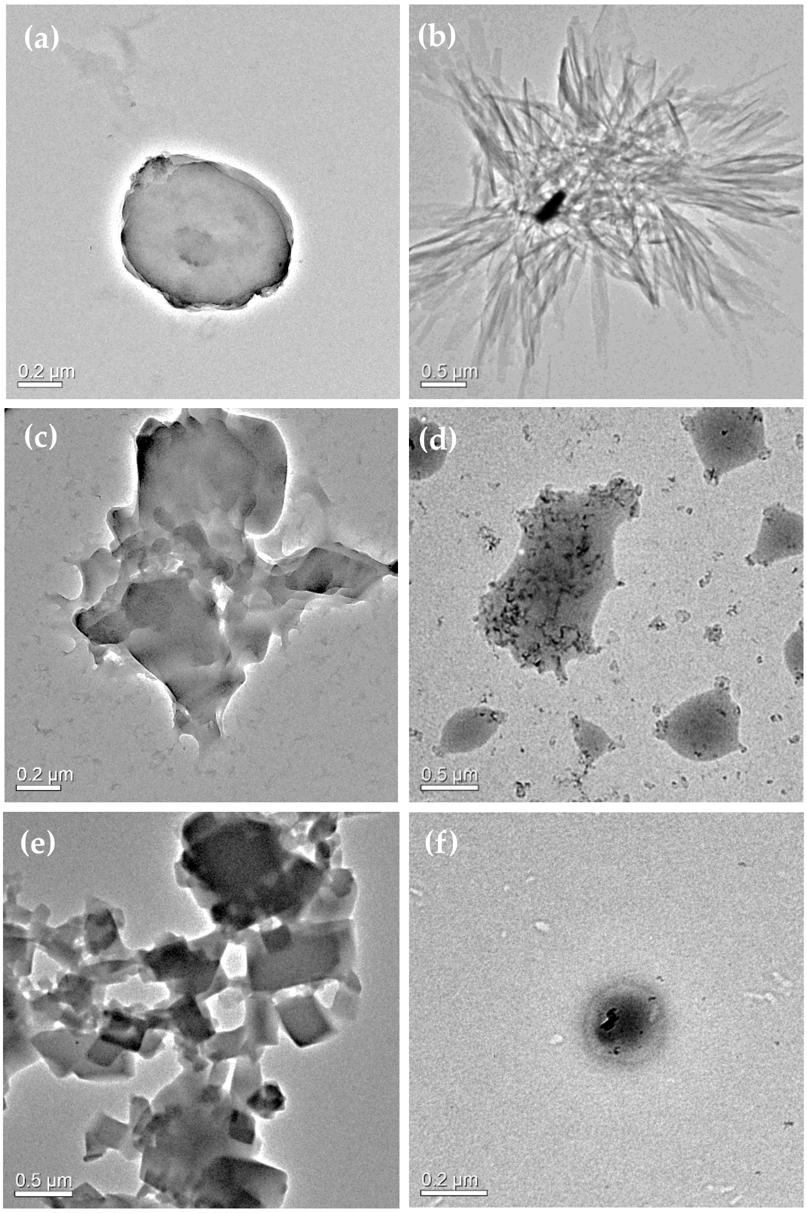

Figure S2. TEM micrographs of (a) processed TAβCD, (b) processed SP, (c) SP1/TAβCD1 PM, (d) spray-dried SP1/TAβCD1 complex, (e) SP1/TAβCD2 PM and (f) spray-dried SP1//TAβCD2 complex.

Table S1. The number average molecular weight (M_n), the weight average molecular weight (M_w) and the dispersity (Đ, M_w/M_n), as determined by ^{1}H-NMR and GPC.

Copolymer	M_n (theoretical) [g mol^{-1}]	M_n (^{1}H-NMR) [g mol^{-1}]	M_n (GPC) [g mol^{-1}]	M_w (GPC) [g mol^{-1}]	Đ (M_w/M_n, GPC)
**mPEG-PCL**	24,000	25,000	19,000	32,600	1.71

Table S2. Equivalent amounts of the different components used for the encapsulation of SP within mPEG-PCL NPs.

Formulation	Equivalent amount used for encapsulation		
	mPEG-PCL [mg]	SP [mg]	TAβCD [mg]
Pristine SP	50	1	-
		2	-
SP1/TAβCD1 DWC		1	8.5
		2	17
SP1/TAβCD2 DWC		1	17
		2	34
SP1/TAβCD1 SD		1	8.5
		2	17
SP1/TAβCD2 SD		1	17
		2	34

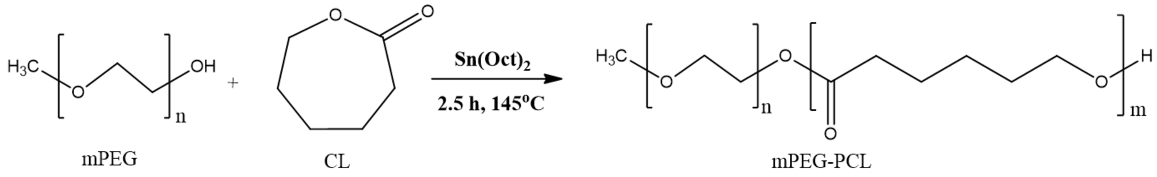

Figure S3. Ring opening polymerization reaction of CL initiated by the terminal hydroxyl group of mPEG with a molecular weight of 4000 g mol^{-1}.

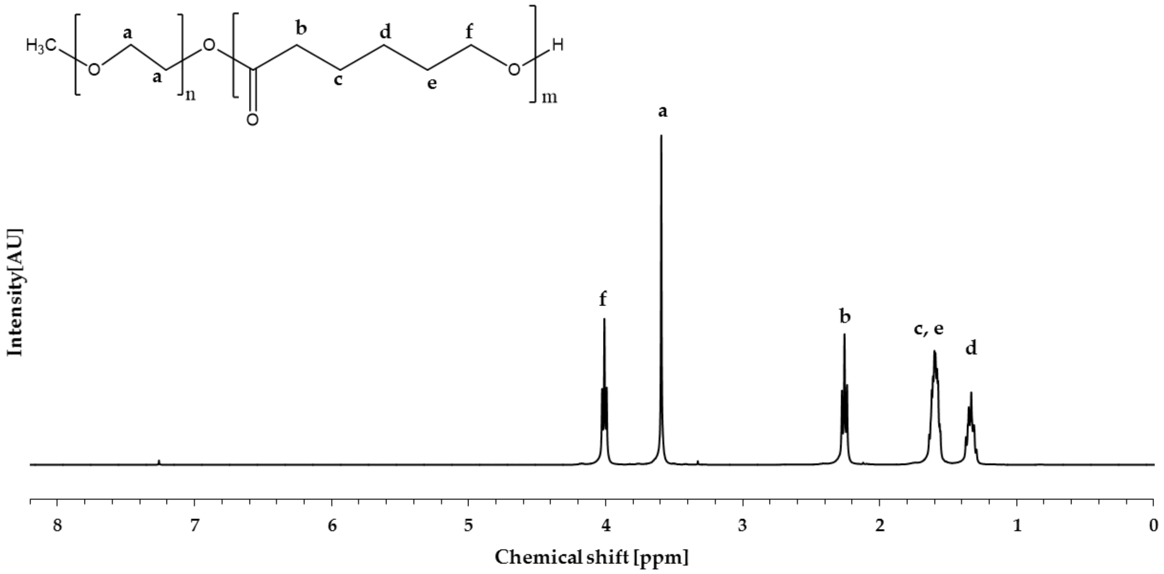

Figure S4. ^{1}H-NMR spectrum of the mPEG-PCL copolymer in $CDCl_3$.

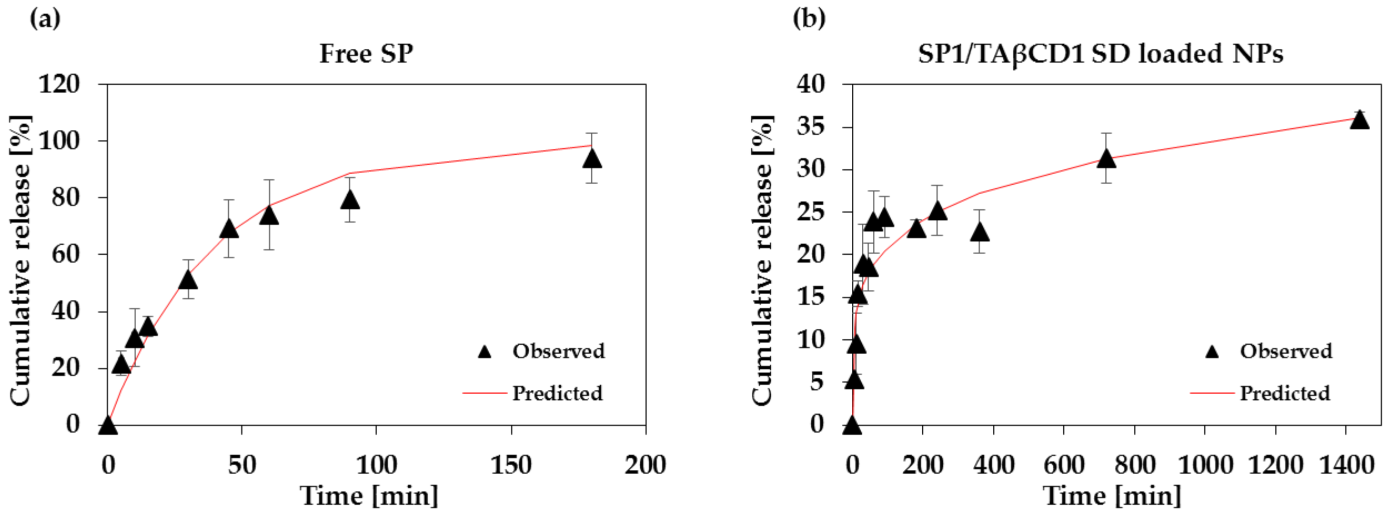

Figure S5. Fitting of average release data of (a) free SP to a first-order kinetics and (b) SP from SP1/TAβCD encapsulated PEG-PCL NPs to the Korsmeyer-Peppas model, as determined with DDSolver Software 1.0 [41].
